# Quantum anomalous Hall heterostructures

**DOI:** 10.1093/nsr/nwy157

**Published:** 2018-12-22

**Authors:** Ke He, Qi-Kun Xue

**Affiliations:** 1 State Key Laboratory of Low Dimensional Quantum Physics, Department of Physics, Tsinghua University, China; 2 Beijing Academy of Quantum Information Sciences, China

The discoveries of the integer and fractional quantum Hall effects in the early 1980s unveiled an exotic world of topological quantum matter [1]. This fascinating world, however, remains largely Platonic in the sense that most of the predicted topological quantum phases are still far away from experimental realization. For a time, almost the only way to find a new topological quantum phase was to search for quantum Hall systems with higher carrier mobilities at lower temperatures and under stronger magnetic fields. In 1988, F. D. M. Haldane conceived a toy model exhibiting the same topological characteristic for its electronic energy band structure—non-zero Chern number (*C*)—to an integer quantum Hall system. It was later known as a quantum anomalous Hall (QAH) insulator [2]. Haldane's work implied the possibility of driving a simple material into topological quantum phase by engineering its electronic band structure. However, it was not until 2005 that a large family of materials capable of embodying various topological quantum phases was found, namely the time-reversal-invariant topological insulator (simply, TI) [3]. The QAH effect was finally experimentally realized in a TI with ferromagnetism introduced by magnetic dopants [4].

The QAH insulator is important not only as a rare topological quantum phase that has been unambiguously experimentally realized, but also as a solid and versatile building block to construct many other topological quantum states. The latter role requires a QAH system to be engineered or incorporated into various kinds of heterostructures; some exciting experimental progress has been made in this area in the past few years.

## AXION INSULATOR

**Figure 1.**
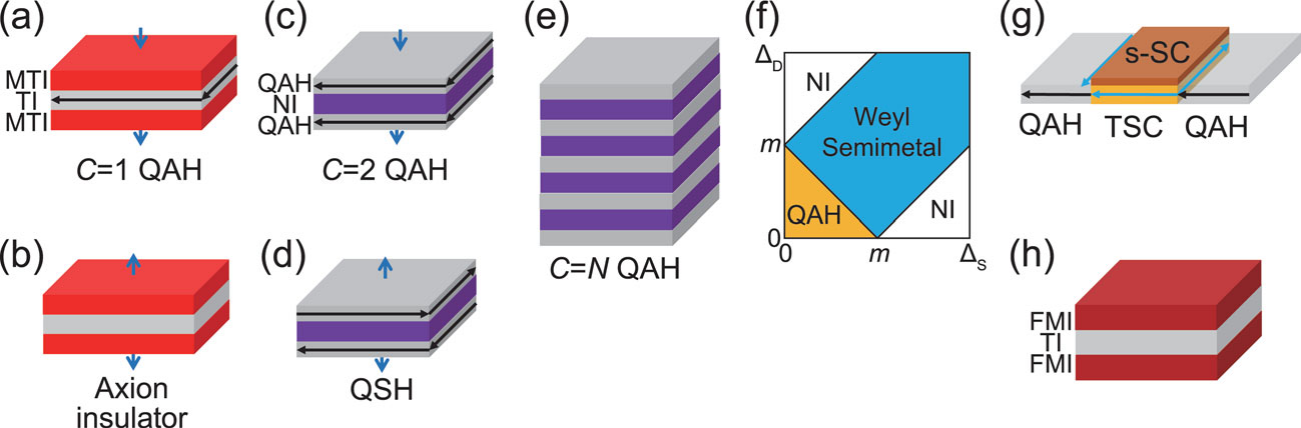
Schematics of several QAH-based heterostructures. (a, b) Magnetic TIs with the magnetizations of the top and bottom surfaces in parallel (a) and anti-parallel (b) configurations, corresponding to *C* = 1 QAH insulator and axion insulator, respectively. The red and gray parts represent magnetic TI (MTI) and TI layers, respectively. The blue arrows indicate the magnetization vectors, and the black lines with arrows represent the chiral edge states. (c, d) QAH bilayers with magnetizations of two QAH layers in parallel (c) and anti-parallel (d) configurations, corresponding to effective *C* = 2 QAH insulator and quantum spin Hall (QSH) insulator, respectively. The gray and violet parts represent QAH and normal insulator (NI) layers, respectively. The blue arrows indicate the magnetization vectors, and the black lines with arrows represent the chiral edge states. (e) QAH multilayer as an effective *C* = *N* QAH insulator. (f) Phase diagram of QAH multilayers. *m* is the magnetic gap size; Δ_D_ is the hybridization between the surface states of neighboring QAH layers; Δ_S_ is the hybridization between the top and bottom surface states of one QAH layer. (Redrawn based on [6].) (g) Transport measurement geometry of observation of the half-quantized plateau of chiral Majorana edge mode. The brown, yellow and gray parts represent s-wave SC, topological SC (induced by superconducting proximity in QAH insulator) and QAH insulator, respectively. The black and blue lines indicate QAH and Majorana edge states, respectively. (h) FMI/TI/FMI sandwich structure expected to show high-temperature QAH effect. The dark-red and gray parts represent FMI and TI layers, respectively.

The QAH phase occurs in a magnetic 3D TI film when the topological surface states of the top and bottom surfaces are gapped by out-of-plane magnetizations along the same direction (Fig. 1a). If the two surfaces have their magnetization vectors opposite to each other, the QAH edge channel is turned off, and the system behaves like a normal insulator (NI) in *dc* transport measurements (Fig. 1b). In *ac* measurements, however, topological magnetoelectric (TME) effects will occur. Unlike the usual magnetoelectric effects, in the TME effects, the electric and magnetic fields are coupled collinearly and related by a coefficient proportional to the fine-structure constant. Such an insulator can be considered as a condensed matter analog of the axion in particle physics and is therefore called an axion insulator [3]. Several experiments have realized the axion insulator structure by choosing different magnetic doping levels or elements near the two surfaces of a QAH film and meanwhile decoupling their magnetizations with a non-magnetically-doped TI layer between them. In these heterostructures, the QAH–NI transitions have been achieved with *dc* measurements, but observation of the TME effects remains a big challenge [5].

## QAH BILAYER AND MULTILAYER

Two identical QAH films spaced by an NI layer are analogous to a *C* = 2 QAH system with two parallel chiral edge channels (Fig. 1c). If the two QAH layers have opposite magnetization vectors, the two chiral edge states have opposite momentum and spin directions, similar to the helical edge states of a quantum spin Hall (QSH) insulator except that they are spatially separated (Fig. 1d). Stacking many QAH layers with NI spacing layers in between leads to an effective high Chern number QAH system (Fig. 1e). Reducing the thicknesses of the QAH layers and NI layers to allow electronic hybridization between the QAH layers, one can obtain a magnetic Weyl semimetal with only one pair of Weyl points (Fig. 1f) [6]. Coulomb interaction may induce more interesting phenomena in the QAH bilayers and multilayers, as it does in quantum Hall bilayers. QAH multilayers have been experimentally realized in superlattices of magnetically doped (Bi, Sb)_2_Te_3_ QAH films and CdSe (0001) NI films grown by molecular beam epitaxy, which paved the way for the search for these topological phases [7].

## CHIRAL TOPOLOGICAL SUPERCONDUCTOR

A QAH insulator acquiring proximity superconductivity from an adjacent *s*-wave superconductor (SC) layer can show the properties of a chiral topological SC [3]. Majorana bound states (MBSs) are expected to appear in the magnetic vortices of the topological SC and can be used to compose topological qubits that are presumably robust against decoherence with topological protection. At the boundary between a chiral topological superconductor and a QAH insulator, there is a dispersive chiral Majorana edge mode that can be considered as half of a chiral QAH edge state. The interferometry of chiral Majorana edge modes is an effective way to detect MBSs and read out topological qubits [8]. Half-quantized plateaus of the two-terminal conductance, a signature of the chiral Majorana edge modes, were reportedly observed in a QAH–Nb heterostructure (Fig. 1g) [9]. A better-defined QAH–SC interface is crucial for further exploration in this direction to avoid disturbance of interface disorders. Recently it was found that an *α*–Sn film of several atomic layers is superconducting and can be epitaxied on Bi_2_Te_3_ family TIs with a PbTe buffer layer, which would make an ideal QAH–SC interface [10].

## FERROMAGNETIC INSULATOR–TOPOLOGICAL INSULATOR HETEROSTRUCTURES

A key issue in the studies of the QAH effect is how to increase its working temperature [11,12]. A TI sandwiched between two ferromagnetic insulator (FMI) layers is believed to be a practical route because of the more ordered structure and potentially higher Curie temperature than magnetically doped TIs (Fig. 1h). Although several works on FMI/TI heterostructures reported ferromagnetic proximity in TIs, the obtained anomalous Hall resistance is far from the quantized value. Interestingly, some studies indicate that an FMI layer inserted into the sub-surface region of a TI can enhance the magnetic gap and the QAH temperature [11]. This is probably because a sub-surface FMI layer interacts more effectively with the topological surface states than an on-surface one [13]. Layered magnetic materials, which have been the subject of intense research interest recently, might be used to realize the more complex FMI/TI heterostructures.

The progress on QAH-based heterostructures provides us with great opportunities to reach various novel topological states of matter experimentally. The explorations in this direction call for more exquisitely designed heterostructures, higher sample quality and better control of the interfaces. With these efforts, the fantastic world of topological quantum matter is getting more and more realistic.
